# Outdoor recess is associated with more positive attitudes toward physical activity among pre-adolescent students and their parents

**DOI:** 10.3389/fspor.2024.1433801

**Published:** 2024-09-16

**Authors:** Getter Marie Lemberg, Merike Kull, Jarek Mäestu, Eva-Maria Riso, Katrin Mägi, Evelin Mäestu

**Affiliations:** Institute of Sport Sciences and Physiotherapy, Faculty of Medicine, University of Tartu, Tartu, Estonia

**Keywords:** outdoor recess, physical activity, attitudes, students, parents, school day

## Abstract

**Introduction:**

School day structure has the potential to increase students’ physical activity (PA) levels and form positive attitudes about PA. Including various PA opportunities and free play possibilities in the school schedule, especially outdoor recess, can improve students’ moderate-to-vigorous PA (MVPA) levels during school time. Therefore, the main aim of the study was to investigate students’ and their parents’ attitudes about outdoor recess and PA opportunities in schools with different recess opportunities.

**Methods:**

Students from grades three to six (9–13-year-olds) and their parents responded to a questionnaire about the effect of outdoor recess and opportunities for PA during the school day. Schools were divided into three groups based on the recess opportunities during the school day: (1) “outdoor recess”, (2) “outdoor recess on some days”, (3) “indoor recess”.

**Results:**

Students and parents of the “outdoor recess” group had significantly more positive attitudes about outdoor recess and PA opportunities in school. Students of the “outdoor recess” group stated being significantly more active during their leisure-time compared to other groups. Parents of the “outdoor recess” group stated that the school has asked their opinion regarding PA opportunities during the school day significantly more compared to the other two groups.

**Discussion:**

These findings emphasize the positive effect outdoor recess can have on students’ PA beliefs and habits. Parents of the “outdoor recess” group also had more positive attitudes toward PA which is important as parents most likely convey their attitudes and beliefs to their children. In addition, involving and informing parents is critical when changing the school schedule and introducing new school culture, to make the changes last.

## 1 Introduction

Physical activity (PA) levels of children and adolescents have been dramatically decreased during COVID-19 pandemic (1). Lower levels of PA and cardiovascular and motor performance have been associated with poorer cognitive function and academic achievement in children (2). Children with higher cardiovascular performance show better cognitive functions which are required inside and outside the classroom, such as executive control, working memory, selective attention, and flexible thinking (3). Moreover, motor skills in childhood can play an important role in cognitive development in the future (4). Therefore, schools are ideal settings for implementing various PA interventions considering the amount of time students spend in school. Implementing PA interventions in schools holds the potential to engage all children from various socio-economic backgrounds (5), which is especially important for students whose only possibility to engage in PA is during school time. In addition, school culture and various activities included in the school schedule can be important factors in developing positive attitudes of PA and the behavioral habits of students (6, 7). However, to change different parts of school schedule and school culture, cooperation with various stakeholders is essential, especially involving parents in the decision-making process (8, 9).

School day structure plays a critical role in increasing students’ school day PA. Including various PA opportunities and free play possibilities in the school schedule, especially outdoor recess, can tremendously improve students’ moderate-to-vigorous PA (MVPA) levels during school time (10, 11). Students can acquire up to one-third of the total school time MVPA during outdoor recess (11). In addition to increased PA levels, spending time outdoors also has a positive effect on students’ cognitive functions, academic behavior, and relationships with other students (4, 12–14). However, to our knowledge, no previous study has explored how different recess opportunities and participation in outdoor recess can affect students’ attitudes of PA and academic behavior.

Systematic reviews show that physical fitness, acute PA, long-term PA interventions, and green time can promote children's cognitive functions and academic behavior (14–17), nevertheless, there is inconsistency in the findings and the impact of various factors related to PA and time spent in green spaces on cognition remain to be explored (15, 17). A meta-analysis by De Greeff et al. (16) highlighted that single-bout PA can restore attention, which is positive for learning processes, but longitudinal PA programs are more likely to improve all domains of executive functions among preadolescent children (18, 19). This emphasizes the importance of implementing changes in school schedules to provide students with more PA opportunities during school time, which in turn increases their PA levels and enhances cognitive functions.

PA during school time, especially outdoors, can have a restoring effect on students’ attention and create a more peaceful learning environment (20–23). Engaging in unstructured outdoor play provides students with a chance to reset their minds and redirect their energies (24). After a walking break outdoors students in Italy reported a reduction in perceived fatigue and a small perceived increase in their attention level in lessons, whereas only 37% of teachers reported an increase in students’ attention levels and 16% stated improved academic proficiency (20). In contrast, Haapala et al. (22) stated that most of the school staff agreed that increased PA during the school day enhanced satisfaction with school and created a more peaceful learning environment. Increased PA at recess is also related to better peer relationships and feelings of belonging (4). During active play, students develop social skills that help to establish and maintain friendships, be active team members, and guide others. Larouche et al. (25) found that every hour per day that was spent outdoors decreased the likelihood of social problems among 7–14-year-olds by 31%. Another study revealed that both parents and teachers emphasized a notable rise in behavioral issues during and after school on days when students had only indoor recess (26).

School is an important factor in developing positive beliefs about PA and PA behaviors, however, a supportive home environment is equally important. The socio-ecological model states that an individual's behavior influences and is influenced by the surrounding environment (27). Parents have the closest connection to their children and therefore can shape their behavior and habits. Consequently, parents’ awareness and knowledge about the positive effects of PA can affect students’ PA habits and behavior, which in turn has an impact on students’ cognitive functions. Pluta et al. (28) found that school-based interventions need to include family and teachers for social support for PA promotion. Moreover, parent engagement has been associated with improved child health outcomes (8, 9), and early support from parents and teachers has been reported as a significant predictor of self-promoted PA (29).

PA habits are mostly developed in childhood and carry on to adult life (30). Therefore, a school environment that supports PA and promotes positive PA habits among students can be a crucial factor in supporting students’ PA behavior during their leisure-time (31, 32). Estonian national program Schools in Motion focuses on promoting a positive PA culture in schools and increasing students’ PA levels by encouraging various PA opportunities throughout the school day (33). Outdoor recess is one of many possibilities that Schools in Motion can implement. The outdoor recess culture is relatively new in Estonia and many schools are at the initial stages of the implementation. Traditionally, in Estonia recesses are spent indoors, and the usual recess length is 10 min. Some schools allow their students to go outside during any recess, however, during the regular 10 min recess, students do not have enough time to go outside and engage in various activities. Furthermore, short recesses do not support the potential for developing positive PA habits and do not conduce to changes in school culture. Therefore, to achieve the aforementioned benefits of outdoor recess, it is important to formally include it in the school schedule to provide students with enough time to be active during outdoor recess and develop positive PA habits. This, in turn, needs changing the structure of the school day for outdoor recess to fulfil its purpose.

Previous research has investigated the effect of various school-related factors, for example, positive PA culture and the existence of various PA facilities in schools, on students’ PA habits and levels (10, 34). However, to our knowledge previous studies have not examined the effect of various recess opportunities on students’ perceptions about PA and their leisure-time PA levels. It is important to investigate the effect of recess opportunities on various PA related factors to provide stakeholders with valuable information on how changing the school day structure can influence students’ PA habits and beliefs, and academic behavior. Therefore, the main aim of this study was to investigate students’ and their parents “perceptions of outdoor recess and its benefits, and PA opportunities in schools with different recess opportunities. Additionally, to investigate whether the recess opportunities are related to students” leisure-time PA.

## 2 Materials & methods

The current article is part of a broader research project that aims to measure students’ PA levels with accelerometers and assess how various schoolyards with different equipment and affordances affect students’ PA levels and preferred activities during outdoor recess (35). Furthermore, the study aims to explore students’ and their parents’ perceptions about outdoor recess and its benefits, and PA opportunities in schools. To focus on how various recess opportunities are associated with students’ and their parents’ opinions about outdoor recess and PA opportunities in school, only part of the questionnaire data is presented in the current manuscript.

### 2.1 Participants

Initially, 19 primary schools in Estonia were contacted and 15 consented to participate in the main research project. All schools were part of the Schools in Motion program, located in different parts of Estonia, and varied in size and school day structure. All students from grades three to six (9–13-years-olds) and one of their legal guardians were invited to participate in the study. In total, 792 students (353 boys, 439 girls; age 10.5 ± 1.2 years) and 759 legal guardians (667 women, 89 men, 3 did not indicate; age 40.1 ± 6.3 years) completed the responded to the questionnaire.

### 2.2 Questionnaire

The questionnaire included questions about demographics, PA opportunities in school, outdoor recess, and leisure-time activities, however not all questions were used in the statistical analysis. As the focus of the current article was to explore students’ and their parents’ opinions about outdoor recess and PA opportunities in schools, only questions relevant to that are presented in this article. The questionnaire was developed and validated by the Schools in Motion program (33). The section about outdoor recess has been previously used by Brustio et al. (20) and was translated into Estonian and modified to fit the context of outdoor recess. All participants were asked to rate how much they agreed with the statements about outdoor recess on five-point Likert scale—(1) fully disagree, (2) disagree, (3) neither agree nor disagree, (4) agree, (5) fully agree. Parents were asked about their attitudes towards the opportunities and promotion of PA in their child's school. They rated the statements on four-point Likert scale—(1) not important at all, (2) not important, (3) important, (4) very important. In addition, students were asked how often they are physically active outdoors during leisure-time, whether (1) rarely, (2) 1–2 times a week, (3) 3–4 times a week, (4) almost every day, (5) every day. For the statistical analysis, responses every day and almost every day, and 1–2 times a week and rarely were grouped together to more clearly present the results of this question.

### 2.3 Data collection

Data collection was performed between September 2021 and December 2022. Before data collection written informed consent was obtained from all participants. Members of the research team handed out the questionnaires at school and participants, both students and parents, completed the questionnaire at home, and returned the completed questionnaire to the research team. All monitoring, collection of data, and analysis were treated anonymously and in line with ethical guidelines. The study was performed in accordance with the Declaration of Helsinki (World Medical Association, 2013), and approved by the Medical Ethics Committee of the University of Tartu, Tartu, Estonia, approval no. 330/T-7.

### 2.4 Data analysis

For the statistical analysis, the schools were divided into three separate groups based on the recess opportunities in schools—(1) “outdoor recess”; (2) “outdoor recess on some days”; (3) “indoor recess”. Schools in the “outdoor recess” group (6 schools; students *n* = 320; parents *n* = 309) had a daily outdoor recess in the school schedule and it was obligatory to go outside during this time. Schools in the “outdoor recess on some days” group (4 schools; students *n* = 229; parents *n* = 222) had one longer active recess every day, on some days of the school week it was an outdoor recess, and on other days it was an active indoor recess where students could access the gym or engage in other physically active activities. Schools in the “indoor recess” group (5 schools, students *n* = 243; parents *n* = 228) did not have outdoor recess as part of the school schedule, however, students were not forbidden to go outside during any recess.

SPSS software for Windows (version 29.0) was used for statistical analysis. The normality of the data was tested using the Shapiro-Wilk test. A Univariate General Linear Model (GLM) was used to examine differences in children's and their parents’ answers between school groups (mea*n* ± SE). All models were adjusted for grade and gender. The distribution of being active (based on questionnaires) during leisure-time between school groups was analysed using Pearson Chi-Square Test. Statistical significance was set at *p* < 0.05.

## 3 Results

### 3.1 Students’ perceptions about outdoor recess, and leisure-time PA

Students’ perceptions about outdoor recess, and their leisure-time PA are presented in Table 1. The “outdoor recess” group had significantly more positive attitudes about outdoor recess compared to “outdoor recess on some days” and “indoor recess” groups (*p* < 0.05), except for the statement about concentration after outdoor recess for the “outdoor recess on some days” group. The “outdoor recess on some days” group had significantly more positive attitudes about concentration after outdoor recess and outdoor recess making the rest of the school day lighter when compared with the “indoor recess” group (*p* < 0.05).

**Table 1:** Students’ perceptions about outdoor recess, and their leisure-time PA (mean ± SE).

	Outdoor recess	Outdoor recess on some days	Indoor recess
In what degree do you agree with the following statements?			
It is easier to concentrate in the lesson after the outdoor recess	3.62 ± 0.06	3.47 ± 0.07	3.18 ± 0.07^b,c^
Participating in the outdoor recess makes the rest of the school day lighter	3.56 ± 0.07	3.17 ± 0.08^b^	2.93 ± 0.08^b,c^
Participating in the outdoor recess has helped to facilitate relationships with my peers	3.62 ± 0.07	3.18 ± 0.08^b^	3.17 ± 0.08^b,c^
Outdoor recess should be all year long	4.02 ± 0.08	3.78 ± 0.09^b^	3.66 ± 0.09^b^
I enjoy being active with my peers during outdoor recess	4.40 ± 0.06	4.05 ± 0.07^b^	4.05 ± 0.07^b^
How often are you physically active outdoors during your leisure-time? Do not include participation in organized sports^a^	2.25 ± 0.05	2.07 ± 0.06^b^	1.94 ± 0.06^b^

Statements are rated on a five-point Likert scale. Adjusted for the grade and gender. ^a^Responses to this question were summarized into three groups (1): rarely or 1–2 times a week (2); 3–4 times a week; (3) almost every day or every day. ^b^Significantly different from “outdoor recess” group. ^c^Significantly different from “outdoor recess on some days” group.

The “outdoor recess” group stated being significantly more active outdoors during their leisure-time compared to “outdoor recess on some days” and “indoor recess” groups (*p* < 0.05) (Table 1). 53.0% of the “outdoor recess” group indicated that they were active outdoors during leisure-time every day or almost every day, whereas the same indicator for “outdoor recess on some days” and “indoor recess” groups was 42.7% and 37.3%, respectively. A similar number of students in all groups stated being active outdoors during leisure-time 3–4 times a week (19.2%–21.4%). Almost half of the students in the “indoor recess” group (43.0%) stated being active outdoors during leisure-time for 2 times a week or less, same indicator for “outdoor recess” and “outdoor recess on some days” group was 27.9% and 35.9%, respectively.

### 3.2 Parents’ perceptions about PA opportunities and their involvement in decision-making

Parents’ perceptions about PA opportunities during school time are presented in Table 2. Compared to the “indoor recess” group parents of “outdoor recess” and “outdoor recess on some days” groups considered it significantly more important that the school provides PA opportunities, including outdoor recess and available equipment, for students during the school day (*p* < 0.05). Parents in the “outdoor recess” group agreed significantly more with statements “It is beneficial to go outside during recess” and that “Being active during recess decreases health risks of students” compared to “outdoor recess on some days” and “indoor recess” groups (*p* < 0.05, Table 2). In addition, 75% of parents in the “outdoor recess” group completely agreed with the statement that “It is beneficial to go outside during recess” compared to 60% and 47% of parents in the “outdoor recess on some days” and “indoor recess” groups and 52% of parents in the “outdoor recess” group completely agreed with a statement that “being active during recess decreases health risks of students” compared to 46% and 41% of parents in the “outdoor recess on some days” and “indoor recess” groups, respectively. Parents of “outdoor recess” and “outdoor recess on some days” groups agreed significantly more with a statement that “Being active during recess promotes a more peaceful learning environment” compared to parents of the “indoor recess” group (*p* < 0.05). Moreover, 47% and 49% of parents in the “outdoor recess” and “outdoor recess on some days” groups, respectively completely agreed with this statement compared to 38% of parents in the “indoor recess” group.

**Table 2:** Parents’ perceptions about PA opportunities during school time (mean ± SE).

	Outdoor recess	Outdoor recess on some days	Indoor recess
How important it is for you that…^a^			
…your child’s school has provided opportunities for students to be active during the school day	3.82 ± 0.02	3.80 ± 0.03	3.72 ± 0.03^c,d^
…your child has outdoor recess every day	3.71 ± 0.03	3.51 ± 0.04^c^	3.25 ± 0.05^c,d^
…your child has an opportunity to be active with their peers during recess	3.81 ± 0.02	3.69 ± 0.03^c^	3.55 ± 0.04^c,d^
…the school has enough equipment (balls, balancing boards, hopscotch etc.) that students can use during recess	3.69 ± 0.03	3.61 ± 0.04	3.50 ± 0.04^c,d^
…school staff encourages students to be active during recess	3.68 ± 0.03	3.62 ± 0.04	3.55 ± 0.04^c^
In what degree do you agree with the following statements?^b^			
It is beneficial for students to go outside during recess	4.71 ± 0.03	4.52 ± 0.04^c^	4.31 ± 0.05^c,d^
Providing opportunities for being active in recess promote students’ relationships with each other	4.35 ± 0.04	4.33 ± 0.05	4.31 ± 0.05
Being active during recess promotes peaceful learning environment	4.38 ± 0.04	4.35 ± 0.05	4.19 ± 0.05^c,d^
Being active during recess decreases health risks of students	4.40 ± 0.04	4.26 ± 0.06^c^	4.25 ± 0.05^c^

^a^ Statements are rated on a four-point Likert scale. ^b^Statements are rated on a five-point Likert scale. ^c^Significantly different from “outdoor recess” group. ^d^Significantly different from “outdoor recess on some days” group.

Parents’ involvement in decision-making about PA opportunities in school is presented in Table 3. Parents of the “outdoor recess” group stated that the school has asked their opinion regarding PA opportunities during the school day significantly more compared to parents of “outdoor recess on some days” and “indoor recess” groups (*p* < 0.5). Fewer parents have cooperated with or supported the school when developing PA opportunities, yet parents of the “outdoor recess” group stated being significantly more involved compared to parents of “outdoor recess on some days” and “indoor recess” groups (*p* < 0.05).

**Table 3:** Parents’ involvement in decision-making about PA opportunities in school.

	Outdoor recess	Outdoor recess on some days	Indoor recess
School has asked my opinion about what could be different in schools, so students could be more active during the school day			
Yes	47.0%	31.7%^a^	17.1%^a,b^
No	53.0%	68.3%^a^	82.9%^a,b^
I have cooperated with or supported the school when developing physical activity opportunities			
Yes	29.0%	9.5%^a^	11.8%^a^
No	71.0%	90.5%^a^	88.2%^a^

^a^ Significantly different from “outdoor recess” group. ^b^Significantly different from “outdoor recess on some days” group.

## 4 Discussion

The main aim of the study was to investigate students’ and their parents’ perceptions of outdoor recess and PA opportunities in schools with different recess opportunities. In addition, to explore if various recess opportunities affect students’ outdoor leisure-time PA. Students and parents of the “outdoor recess” group had the most positive perceptions about outdoor recess and PA opportunities provided in schools. Students in the “outdoor recess” group stated being the most active outdoors out of the three groups during their leisure-time. Additionally, parents of the “outdoor recess” group had the most involvement and participation in the decision-making process about PA opportunities in school.

In the current study, recess opportunities were related to students’ and parents’ perceptions about outdoor recess and PA opportunities in school with the “outdoor recess” group having significantly more positive perceptions. Students in the “outdoor recess” and “outdoor recess on some days” groups stated significantly better concentration in lessons after outdoor recess and that outdoor recess made the rest of the school day feel lighter compared to the “indoor recess” group. Even though students in all three groups reported enjoying being active with their peers during outdoor recess, the mean answer for students in the “outdoor recess” group was 4.40 ± 0.06, which was relatively high on a 5-point Likert scale and was significantly higher than in other two groups (*p* < 0.05). Previous research has also found that recess is positively related to peer relationships and on days of outdoor recess, students had less social problems with each other (4, 26). In addition, outdoor recess can have a positive effect on students’ attention and concentration; however, available data is contradictory depending on the type of schoolyard. Van Dijk-Wesselius et al. (21) found that after the greening of the schoolyards students’ attention restoration score improved more compared to the control group whose schoolyard did not go through the greening process. Similarly, Amicone et al. (36) found that students’ attention improved after spending the recess in green schoolyard and decreased after spending recess in the artificial schoolyard. One of the most common reasons that school staff and parents state not including outdoor recess in the school schedule is due to weather and the appropriate clothing. However, the results of the current study indicated that students in the “outdoor recess” group agreed that outdoor recess should be all year long. This result emphasizes that when the outdoor recess culture has already been formed in school, the weather and the clothing are not a hindrance anymore, and students willingly go outside throughout the year with every weather.

Students spend the majority of their day in school; therefore, school can be an important influencer of students’ PA habits. Students in the “outdoor recess” group stated being significantly more active outdoors during their leisure-time compared to the other two groups. Schools that recognize the importance of the PA, provide their students with various PA opportunities throughout the school day which in turn can help to form positive PA habits which transfer to students’ everyday life and their leisure-time. Therefore, students in the “outdoor recess” group might estimate their leisure-time outdoor PA higher than students in other two groups. However, students self-reported their leisure-time outdoor PA levels and objective data might not support it, as both children and adults tend to overestimate their PA levels when asked to self-report (37). Hence, further research should objectively measure leisure-time PA levels to assess outdoor recess’ relation to leisure-time PA levels.

Changing the schedule of the school day by providing various PA opportunities during the school day, including outdoor recess, increases students’ school time PA levels which in turn increase their daily MVPA (10, 11). The systematic review by Sanches & Gallego (10) found that the school time MVPA contributes to more than 40% of the daily MVPA. These results clearly emphasize the importance of outdoor recess and a school schedule that involves various possibilities for PA to increase students’ MVPA levels. This is critical for students who do not have the possibility to be active after school, especially outdoors.

Family has an important role in shaping children's PA habits and beliefs (27). If parents have positive perceptions about PA, they are more likely to convey these beliefs and perceptions to their children. In the current study, parents of the “outdoor recess” group had significantly more positive perceptions of outdoor recess and its benefits compared to other two groups. However, parents of the “outdoor recess on some days” group had significantly more positive perceptions and beliefs compared to the “indoor recess” group. Parents of the “outdoor recess” and “outdoor recess on some days” groups stated that the school has asked their opinion about developing PA opportunities in school significantly more compared to the “indoor recess” group. Possibly schools that provide outdoor recess every day or on some days have informed parents more about the benefits of outdoor recess and the overall PA, and therefore have more positive perceptions about outdoor recess, PA, and its benefits. Hence, involving and informing parents is critical when changing the school schedule and introducing new school culture, to make the changes last. Previous studies have also found that parent engagement and support were associated with improved child health outcomes and were predictors of self-promoted PA (8, 9, 29).

Some strengths and limitations of the current study should be addressed. The strength of this study is a relatively large sample of students and parents who participated in the study. Furthermore, the study included different schools with various recess opportunities. One limitation of the study is that students subjectively rated their leisure-time outdoor PA, and outdoor recess’ effect on concentration and making the school day feel lighter. In addition, some parents might not be informed about everything that happens at the school. Also, all participating schools have somewhat different schoolyards, which can affect students’ opinions about outdoor recess and the activities they can participate in during outdoor recess.

In conclusion, the results of this study indicate that schools that provide the possibility to go outside during the school day can have a positive impact on students’ perceptions and beliefs about outdoor recess and PA. Therefore, the school schedule should be changed in a way that provides students with the opportunity to go outside and be active in the schoolyard during school time. Initially, outdoor recess can be provided on some days of the school week. However, to maximize the benefits of outdoor recess, eventually students should have the possibility to participate in outdoor recess every day all year long as it improves their attention, academic behavior, relationships with peers, and attitudes about PA. In addition, involving more parents in the decision-making process about PA opportunities in school helps to modify parents’ perceptions and beliefs about PA opportunities in school. Students and their parents in schools with outdoor recess every day had the most positive perceptions about outdoor recess and PA opportunities in school. In order to change the behavior, it is essential to change the attitudes first. Therefore, the results of this study emphasize the impact of outdoor recess on students’ perceptions and beliefs which in turn can also influence their PA levels. Furthermore, informing parents about the positive impacts of PA and outdoor recess and involving them in decision-making processes in school is critical in changing the school culture and making the changes last.

## Data Availability

The datasets presented in this article are not readily available because data cannot be shared publicly as our participants and their legal guardians were not asked to consent to data-sharing outside of our research group. Availability of data has to be in accordance with the Medical Ethics Committee of the University of Tartu, Tartu, Estonia. Requests to access the datasets should be directed to Evelin Mäestu, evelin.maestu@ut.ee.
